# Improvement of Severe COVID-19 in an Elderly Man by Sequential Use of Antiviral Drugs

**DOI:** 10.1155/2020/8814249

**Published:** 2020-09-05

**Authors:** Masakiyo Yatomi, Tomonori Takazawa, Kunio Yanagisawa, Masafumi Kanamoto, Yusuke Matsui, Hiroyuki Tsukagoshi, Nobuhiro Saruki, Shigeru Saito, Yutaka Tokue, Toshitaka Maeno

**Affiliations:** ^1^Department of Allergy and Respiratory Medicine, Integrative Center of Internal Medicine, Gunma University Hospital, 3-39-15 Showa-machi, Maebashi, Gunma, Japan 371-8511; ^2^Intensive Care Unit, Gunma University Hospital, 3-39-15 Showa-machi, Maebashi, Gunma, Japan 371-8511; ^3^Infection Control and Prevention Center, Gunma University Hospital, 3-39-15 Showa-machi, Maebashi, Gunma, Japan 371-8511; ^4^Gunma Prefectural Institute of Public Health and Environmental Sciences, 378 Kamioki-machi, Maebashi, Gunma, Japan 371-0052

## Abstract

Although a variety of existing drugs are being tested for patients with coronavirus disease 2019 (COVID-19), no efficacious treatment has been found so far, particularly for severe cases. We report successful recovery in an elderly patient with severe pneumonia requiring mechanical ventilation and extracorporeal membrane oxygenation (ECMO). Despite administration of multiple antiviral drugs, including lopinavir/ritonavir, chloroquine, and favipiravir, the patient's condition did not improve. However, after administration of another antiviral drug, remdesivir, we were able to terminate invasive interventions, including ECMO, and subsequently obtained negative polymerase chain reaction results. Although further validation is needed, remdesivir might be effective in treating COVID-19.

## 1. Introduction

A novel coronavirus designated SARS-CoV-2, which is the cause of COVID-19, first appeared in Wuhan, a city in Central China, in December 2019, and has led to a pandemic that is still expanding [1–3]. Since the risk of severe symptoms is very high in elderly patients with COVID-19, often tending to be critical, management of these patients is crucial. Many attempts have been made to identify drugs that might be effective in treating COVID-19. Consequently, some existing drugs, including anti-HIV, anti-influenza, and antimalarial agents, have been given to patients as off-label use for the treatment of COVID-19 [4, 5]. One antiviral drug, remdesivir, which was developed for the treatment of Ebola hemorrhagic fever [6] and was previously found to be highly effective in the control of SARS-CoV-2 in vitro [7], showed promising preliminary results in a recent report [8]. Although many trials using promising antiviral drugs are currently ongoing in parallel all over the world, most of them are primarily aimed at mild or moderately ill patients. Therefore, little is known about the effectiveness of these drugs in severe clinical cases of COVID-19. Further, the proper administration method, including drug dose and duration, and drug safety information, such as side effects, are unknown. Our current report presents a case of an elderly man with severe COVID-19 who improved with sequential use of antiviral drugs, including remdesivir. The clinical course of this patient might provide useful information that could enable physicians to determine which agent can be used in such severe cases of COVID-19.

## 2. Case Report

Written informed consent for the off-label use of drugs and publication of this report was obtained from the patient and his family. The patient was an 82-year-old man who was a passenger on the Diamond Princess cruise ship, which experienced an outbreak of novel coronavirus (SARS-CoV-2). Prior to the off-label use of the antiviral drugs (lopinavir/ritonavir, chloroquine, and favipiravir) and use of remdesivir, which is an unapproved drug in Japan, all the drugs were reviewed and approved by the ethics committee of our hospital. Remdesivir was obtained from the drug manufacturer (Gilead Science, Inc., Foster City, CA, USA) through a Compassionate Use Program [8, 9].

**Figure 1:**
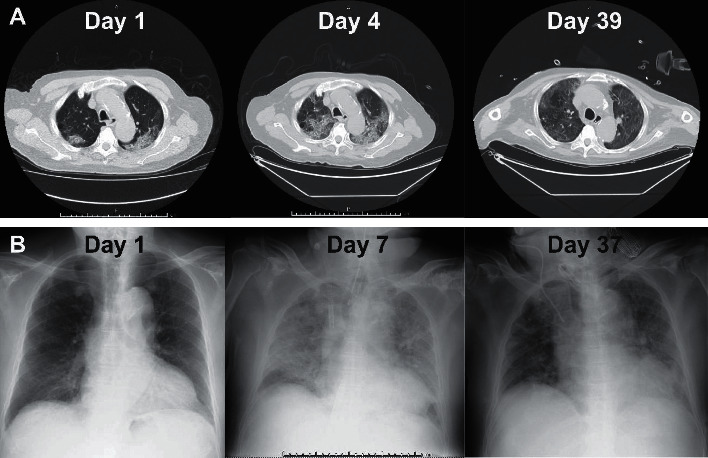
Radiological findings in the patient. (a) Transverse chest CT images on days 1, 4, and 39. Bilateral infiltrative shadows and ground-glass lesions were apparent, which deteriorated on day 4. On day 39, ground-glass lesions decreased and lung aeration improved. (b) Chest radiographs showing changes in lung opacity over time. An endotracheal tube and blood delivery cannulas for ECMO are visible in the image taken on day 7. These were removed, and a tracheostomy tube is visible in the image taken on day 37.

**Figure 2:**
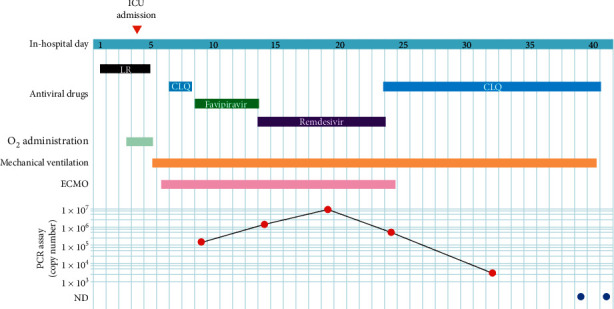
Clinical course of treatment. The viral load peaked on the 19^th^ day during the administration of remdesivir and then started to decrease. Red and blue circles indicate positive and negative results on PCR assay, respectively. LR, lopinavir/ritonavir; CLQ, chloroquine; ECMO, extracorporeal membrane oxygenation; PCR, polymerase chain reaction.

The patient tested positive for SARS-CoV-2 by real-time reverse transcription-polymerase chain reaction (rRT-PCR) assay while on board the ship. Two days later, he was transferred to the infectious disease ward at our hospital. He had a history of bronchial asthma, hypertension, and dyslipidemia and showed no respiratory symptoms including cough, sputum, and dyspnea at the time of admission. His vital signs, except blood pressure (165/94 mmHg), were within their normal ranges at admission. Computed tomography (CT) scan and chest radiographs showed bilateral, subpleural ground-glass opacities in both lungs (Figure 1). Blood tests showed almost normal results on admission (Table S1). Both urinary pneumococcal antigen and urinary legionella antigen were negative. Simple inspection kit results were negative for both influenza virus types A and B. Oral administration of lopinavir/ritonavir, an anti-human immunodeficiency virus (HIV) drug, and ceftriaxone were commenced.  Figure 2 illustrates the course of use of therapeutic drugs and medical devices.

On day 2, he had no noticeable symptoms other than dry cough. On day 3, however, he developed appetite loss and a fever of 38°C. SpO_2_ decreased to 90% on room air. Oxygen administration via a nasal cannula was started that night. However, his respiratory condition further deteriorated on day 4, and he was unable to maintain an SpO_2_ of 90% even with administration of 2 L/min oxygen. CT scan clearly showed exacerbation of the pulmonary pathology compared to the previous scan, with infiltration and reticular shadows appearing in wide areas of both lung fields (Figure 1). He was transferred to the intensive care unit (ICU), where a blood test showed worsening of inflammatory findings (Table S1).

Poor oxygenation continued after ICU admission, with an SpO_2_ of 90–95% even with oxygen administration of 10 L/min using a reservoir mask. Tracheal intubation was performed due to further worsening of oxygenation at midnight on day 5. We initiated mechanical ventilation in the pressure-controlled mode. Sedation with propofol and fentanyl and administration of dopamine and noradrenaline were commenced after intubation. The antibiotic was changed to meropenem, and oral administration of lopinavir/ritonavir was discontinued. In the afternoon of day 6, we decided to introduce veno-venous extracorporeal membrane oxygenation (VV-ECMO) because his oxygenation could not be maintained with the assistance of mechanical ventilation alone. On day 7, administration of chloroquine, which has been reported to have an inhibitory effect on coronavirus, was commenced and was continued for two days until favipiravir became available. On day 9, the administration of favipiravir, which was developed for treating RNA viruses including influenza virus, was started. On the first day, 1600 mg was administered twice, and thereafter, 600 mg was administered through a gastric tube twice per day for 5 days, as instructed in the package insert. However, even after five days of favipiravir treatment in this case, PCR assay using sputum collected from the lower respiratory tract continued to show a positive result (Figure 2, Table S2).

Administration of remdesivir was started on day 14, with an initial IV loading dose of 200 mg, followed by 100 mg IV once daily for 9 days. ECMO was terminated on day 24 with improvement in his symptoms. The administration of chloroquine was also resumed on day 24. On day 32, tracheostomy was performed. Through this period, we repeatedly performed sputum testing using PCR assay, which was established broadly in Japan [10]. Briefly, RNA was extracted using the QIAamp Viral RNA Mini kit (QIAGEN, Valencia, VA) and the primer set that targeted the specific gene of SARS-CoV-2 coding N protein (N2). The results were validated by comparing the results to the synthesized RNA of an internal control, following which cycle threshold (Ct) values were determined. Details of the sets are shown in the supplemental methods. Ultimately, we confirmed a negative PCR result 41 and 43 days after the first positive result (Figure 2; Table S2) and weaned the patient off mechanical ventilation. Currently, although he has recovered from the life-threatening condition, he requires continued treatment for several complications, namely, bacterial infection and liver dysfunction, which is suspected as being induced by one of the therapeutic drugs.

## 3. Discussion

In this case, we evaluated the effects of antiviral drugs in an elderly patient with COVID-19. Although we initially struggled with treatment even after administration of multiple promising drugs, including lopinavir/ritonavir, chloroquine, and favipiravir, both clinical symptoms and viral load estimated by PCR improved after the administration of remdesivir.

Lopinavir/ritonavir were first administered for a total of four days. However, deterioration of clinical symptoms, such as a decrease in pulmonary oxygenation, could not be prevented during its administration. Moreover, the appearance of diarrhea and inability to eat seemed to be side effects of lopinavir/ritonavir, suggesting their poor tolerability by elderly patients. Considering the results of PCR assay, the effect of lopinavir/ritonavir seemed to be poor. Indeed, a recent study has not shown significant efficacy of these drugs and has suggested that lopinavir/ritonavir might not be recommended for COVID-19 treatment [11].

Next, we administered chloroquine twice, on days 6-7 and on days 24–40. Since we expected that the effect of favipiravir would outweigh that of chloroquine, we used chloroquine as a substitute until favipiravir became available. It is unclear whether chloroquine was effective or not because of the short period of the first administration and the fact that the patient was already on a recovery course in the second period. Furthermore, chloroquine was suspected to be a cause of liver dysfunction and had to be discontinued in the second period.

The third drug, favipiravir, is an antiviral drug with activity against many RNA viruses. It was developed by Toyama Chemical Co. Ltd. (Fujifilm group) of Japan and was approved for stockpiling against influenza pandemics. Favipiravir is currently being studied in China for the experimental treatment of the emergent COVID-19 (ChiCTR2000029600 and ChiCTR2000029548). Since the patient's respiratory condition did not worsen during the administration of favipiravir, it might have a modest virus suppression effect. Although favipiravir did not cause any adverse events unlike lopinavir/ritonavir and chloroquine, a five-day course, as has been prescribed for the treatment of influenza virus infection, might be too short to achieve sufficient effects. Japanese clinicians have reported that they have given favipiravir to a patient with severe fever with thrombocytopenia syndrome and that its duration can be extended up to 14 days [12]. According to their experience, favipiravir can be given earlier or for more days to suppress the replication of SARS-CoV-2.

The fourth drug, remdesivir, is an investigational broad-spectrum antiviral agent. It was developed by Gilead Sciences Inc. and previously tested in humans with Ebola virus disease [6]. It has also shown promise in animal models of Middle East respiratory syndrome (MERS) and severe acute respiratory syndrome (SARS), which are caused by other coronaviruses [13]. We confirmed improvement in the patient's symptoms, clinical data, and viral load, as estimated by PCR assay, especially after the administration of remdesivir, allowing for withdrawal from ECMO. Although it is difficult to conclude anything from this single case, we believe that remdesivir might have significant effects in eliminating SARS-CoV-2 [14]. A recent report showing improvement in clinical symptoms in 68% of patients receiving remdesivir and a total mortality rate of 13% over a median follow-up period of 18 days supports our belief [8]. Fortunately, there were no apparent complications due to remdesivir, as was also reported in a previous trial of the drug for Ebola hemorrhagic fever [6]. In the present patient, only remdesivir was given intravenously among the antiviral agents. This could have resulted in quick elevation of plasma drug concentration without the first-pass effect. Furthermore, since most patients with COVID-19 who are in a severe condition cannot swallow tablets orally, oral antiviral drugs need to be crushed and given via the feeding tube. This could adversely influence the stability of the drug. Hence, intravenous administration of remdesivir directly into the patient's blood circulation without loss of drug dose and activity might be advantageous. Administration of remdesivir for ten days, which was longer than the duration of administration of favipiravir, might have contributed to its superior outcome.

Besides antivirals, other drugs and treatments, such as antibacterial drugs, blood transfusions, artificial respiration, and ECMO, also contributed to achieving significant recovery of the patient. Close collaboration between experts from multiple fields led to the successful management of this difficult case.

In conclusion, we report an elderly man with severe COVID-19 who was successfully treated by sequential administration of multiple antiviral agents, including remdesivir. Our experience would be valuable for all clinicians treating similar patients.

## Data Availability

Data relevant to this case report are not available for public access because of patient privacy concerns, but are available from the corresponding author on reasonable request.

## Supplementary Materials

Supplementary Materials This article includes two supplementary tables and supplementary methods.

## References

[1] WHO (2020). *WHO Director-General’s opening remarks at the media briefing on COVID-19-25 March 2020*.

[2] Huang C., Wang Y., Li X. (2020). Clinical features of patients infected with 2019 novel coronavirus in Wuhan, China. *The Lancet*.

[3] Wang D., Hu B., Hu C. (2020). Clinical characteristics of 138 hospitalized patients with 2019 novel coronavirus-infected pneumonia in Wuhan, China. *JAMA*.

[4] Li G., Clercq E. D. (2020). Therapeutic options for the 2019 novel coronavirus (2019-nCoV). *Nature Revies Drug Discovery*.

[5] Zhang L., Liu Y. (2020). Potential interventions for novel coronavirus in China: a systematic review. *Journal of Medical Virology*.

[6] Mulangu S., Dodd L. E., Davey R. T. (2019). A randomized, controlled trial of Ebola virus disease therapeutics. *New England Journal of Medicine*.

[7] Wang M., Cao R., Zhang L. (2020). Remdesivir and chloroquine effectively inhibit the recently emerged novel coronavirus (2019-nCoV) in vitro. *Cell Research*.

[8] Grein J., Ohmagari N., Shin D. (2020). Compassionate use of remdesivir for patients with severe Covid-19. *New England Journal of Medicine*.

[9] Gilead Sciences I (2020). t. *Individual access to investigational medicines intended to reat serious diseases*.

[10] Shirato K., Nao N., Katano H. (2020). Development of genetic diagnostic methods for novel coronavirus 2019 (nCoV-2019) in Japan. *Japanese Journal of Infectious Diseases*.

[11] Cao B., Wang Y., Wen D. (2020). A trial of lopinavir-ritonavir in adults hospitalized with severe Covid-19. *The New England Journal of Medicine*.

[12] Yasukawa M. (2017). *Development of Effective Treatment for Severe Febrile Thrombocytopenia Syndrome*.

[13] NIH (2020). *NIH Clinical Trial of Remdesivir to Treat COVID-19 Begins*.

[14] Holshue M. L., DeBolt C., Lindquist S. (2020). First case of 2019 novel coronavirus in the United States. *The New England Journal of Medicine*.

