# Supplementary material for: Improvement of Severe COVID-19 in an Elderly Man by Sequential Use of Antiviral Drugs
Source: Case Rep Infect Dis. 2020 Sep 5;2020:8814249. doi: 10.1155/2020/8814249 (PMC7475736; doi:10.1155/2020/8814249)
Supplement: Supplementary Materials — This article includes two supplementary tables and supplementary methods. [file 8814249.f1.zip › 8814249.f1/Supplementary methods.docx]

Supplementary methods

Sputum samples were stored at 4°C until further use. For testing, the sputum sample was thoroughly mixed with BD Universal Viral Transport Medium (BD, USA), following which it was centrifuged at 3000 x g for 15 min at 4°C, and the supernatant was used for experiments. Viral nucleic acid was extracted using the QIAamp Viral RNA Mini kit (Qiagen, Valencia, CA, USA) following the manufacturer’s instructions.

Control standard RNA was donated by the National Institute of Infectious Diseases. Quantitative real-time PCR (qRT-PCR) was performed with a 7500 Fast Real-Time PCR System (Thermo Fisher Scientific, Waltham, MA, USA) as follows: reverse transcription (50°C for 30 min) and denaturation at 95°C for 15 min to activate DNA polymerase, followed by 45 cycles of amplification with denaturation at 95°C for 15 sec, and annealing and extension at 60°C for 1 min using the QuantiTect Probe RT-PCR Kit (QIAGEN). Amplified data were collected and analyzed using the 7500 fast System Software v2.0.6 (Thermo Fisher Scientific).

The primers and probes used in RT-PCR are listed in the following table.

Table

|  | Name | Sequence (5’ to 3’) | Nucleotide position |
| --- | --- | --- | --- |
| Primer F | NIID_2019-nCOV_N_F2 | AAATTTTGGGGACCAGGAAC | 29142-29161 |
| Primer R | NIID_2019-nCOV_N_R2 | TGGCAGCTGTGTAGGTCAAC | 29299-29280 |
| Probe | NIID_2019-nCOV_N_P2 | FAM-ATGTCGCGCATTGGCATGGA-BHQ | 29239-29258 |
