# Supplementary material for: Improvement of Severe COVID-19 in an Elderly Man by Sequential Use of Antiviral Drugs
Source: Case Rep Infect Dis. 2020 Sep 5;2020:8814249. doi: 10.1155/2020/8814249 (PMC7475736; doi:10.1155/2020/8814249)
Supplement: Supplementary Materials — This article includes two supplementary tables and supplementary methods. [file 8814249.f1.zip › 8814249.f1/Table S1 Clinical laboratory results.docx]

Table S1 Clinical laboratory results

| Parameters | | Reference range | Day 1 | Day 4 | Day 6 | Day 8 | Day 10 | Day 16 | Day 22 | Day 24 | Day 32 | Day 41 |  |
| --- | --- | --- | --- | --- | --- | --- | --- | --- | --- | --- | --- | --- | --- |
| White cell count (per μL) | | 4000-9600 | 4000 | 4600 | 9600 | 6800 | 10200 | 10400 | 6200 | 5700 | 13500 | 17700 |  |
| Red cell count (per μL) | | 4200000-5700000 | 5040000 | 487000 | 4470000 | 3500000 | 3710000 | 4390000 | 4480000 | 4620000 | 4240000 | 4110000 |  |
| Percentage of neutrophils (%) | | 42.2-73.2 | 74 | 85 | 95 | 92 |  |  | 63 |  | 92 | 88 |  |
| Percentage of lymphocytes (%) | | 20.1-47.3 | 18 | 11 | 3 | 5 |  |  | 22 |  | 3 | 3 |  |
| Platelet count (per μL) | | 160000-350000 | 117000 | 106000 | 119000 | 83000 | 62000 | 86000 | 91000 | 100000 | 219000 | 216000 |  |
| Hemoglobin (g/dL) | | 13.2-17.3 | 16.0 | 15.2 | 14.2 | 11.0 | 11.2 | 13.3 | 13.4 | 14.0 | 13.1 | 12.8 |  |
| Hematocrit (%) | | 40-52 | 48 | 45 | 41.7 | 33 | 34 | 40.1 | 41.6 | 42.9 | 40.2 | 39.4 |  |
| Sodium (mmol/L) | | 137-145 | 135 | 138 | 140 | 143 | 145 | 139 | 144 | 142 | 140 | 140 |  |
| Potassium (mmol/L) | | 3.5-4.8 | 3.0 | 2.7 | 3.4 | 3.5 | 3.2 | 4.5 | 3.8 | 3.9 | 4.4 | 3.0 |  |
| Chloride (mmol/L) | | 100-107 | 98 | 100 | 103 | 108 | 109 | 105 | 109 | 108 | 102 | 106 |  |
| Calcium (mmol/L) | | 8.9-10.5 | 9 | 8.2 | 7.4 | 6.7 | 7.4 | 9.7 | 10.1 | 9.9 | 9.6 | 8.6 |  |
| Glucose (mmol/L) | | 80-110 | 114 | 180 | 151 | 157 | 185 | 143 | 119 | 119 | 159 | 158 |  |
| Blood urea nitrogen (mg/dL) | | 8-20 | 20 | 25 | 19 | 33 | 24 | 65 | 55 | 40 | 35 | 30 |  |
| Creatinine (mg/dL) | | 0.65-1.07 | 1.02 | 1.07 | 0.98 | 0.8 | 0.69 | 1.17 | 0.96 | 0.89 | 0.84 | 0.59 |  |
| Total protein (g/dL) | | 6.3-7.9 | 7.2 | 6.2 | 5.1 | 4.2 | 4.6 | 7.2 | 7.0 | 6.5 | 6.9 | 7.0 |  |
| Alanine aminotransferase (IU/L) | | 8-42 | 33 | 38 | 27 | 37 | 52 | 32 | 27 | 20 | 74 | 116 |  |
| Aspartate aminotransferase (IU/L) | | 13-33 | 31 | 49 | 37 | 78 | 66 | 29 | 33 | 25 | 42 | 464 |  |
| Alkaline phosphatase (U/L) | | 115-359 | 137 | 106 | 102 | 111 | 128 | 170 | 185 | 176 | 232 | 312 |  |
| Fibrinogen (mg/dL) | | 150-330 | 425 |  | 515 | 476 | 459 | 425 | 290 | 221 | 425 | 459 |  |
| Lactate dehydrogenase (U/L) | | 119-229 | 235 | 347 | 521 | 630 | 665 | 660 | 545 | 521 | 270 | 359 |  |
| Prothrombin ratio (%) | | 70-130 | 96 | 90 | 96 | 97 | 92 | 94 | 83 | 79 | 84 | 76 |  |
| D-dimer (μg/mL) | 0.0-1.0 | | 0.5 | 0.5 | 9.3 | 2.5 | 9.0 | 1.8 | 18.9 | 4.9 | 7.2 | 4.8 |  |
| Creatine kinase (U/L) | | 62-287 | 229 | 758 | 359 | 2631 | 529 | 75 | 47 | 36 | 10 | 37 |  |
| C-reactive protein (mg/dL) | | < 0.1 | 2.3 | 5.7 | 15.6 | 15.8 | 14.5 | 15.5 | 7.3 | 6.9 | 11.0 | 6.0 |  |
| Blood gas analysis | |  |  |  |  |  |  |  |  |  |  |  |  |
| Fraction of inspired oxygen | | | Room air | NC | 0.55 | 0.30 | 0.30 | 0.25 | 0.21 | 0.21 | 0.35 | 0.30 |  |
|  | | |  | 2 L/min |  |  |  |  |  |  |  |  |  |
| pH | | 7.35-7.45 | 7.47 | 7.56 | 7.49 | 7.44 | 7.42 | 7.40 | 7.37 | 7.42 | 7.47 | 7.46 |  |
| pCO_2_ (mmHg) | | 35-45 | 35.7 | 29.7 | 38.9 | 41.0 | 46.4 | 32.5 | 43.3 | 39.0 | 36.8 | 34.5 |  |
| pO_2_ (mmHg) | | 75-100 | 69.7 | 53.4 | 51.0 | 70.1 | 64.3 | 68.3 | 54.5 | 61.8 | 79.4 | 86.2 |  |
| Base excess | | -3-+3 | 1.6 | 3.5 | 6.1 | 3.5 | 4.9 | -5.6 | -0.2 | 0.7 | 3.1 | 0.8 |  |
| Lactate | |  |  |  | 1.4 | 1.2 | 1.1 | 0.8 | 0.8 | 0.7 | 1.2 | 1.2 |  |

A blank indicates no data. Abbreviations: NC, nasal cannula; pCO_2_, partial pressure of carbon dioxide; pO_2_, partial pressure of oxygen
