# Supplementary material for: Improvement of Severe COVID-19 in an Elderly Man by Sequential Use of Antiviral Drugs
Source: Case Rep Infect Dis. 2020 Sep 5;2020:8814249. doi: 10.1155/2020/8814249 (PMC7475736; doi:10.1155/2020/8814249)
Supplement: Supplementary Materials — This article includes two supplementary tables and supplementary methods. [file 8814249.f1.zip › 8814249.f1/Table S2.docx]

Table S2 Results of real-time reverse transcription-polymerase chain reaction testing for the novel coronavirus (SARS-CoV-2)

|  | Day 9 | Day 14 | Day 19 | Day 24 | Day 32 | Day 39 | Day 41 |
| --- | --- | --- | --- | --- | --- | --- | --- |
| Copy number (copies/mL) | 1.5×10^5^ | 1.4×10^6^ | 9.2×10^6^ | 5.0×10^5^ | 2.9×10^3^ | ND | ND |

ND: not detected
